# Multicriteria Decision Analysis and Grouping of Analytical Procedures for Phthalates Determination in Disposable Baby Diapers

**DOI:** 10.3390/molecules26227009

**Published:** 2021-11-19

**Authors:** Magdalena Fabjanowicz, Justyna Płotka-Wasylka, Marek Tobiszewski

**Affiliations:** 1 Department of Analytical Chemistry, Chemical Faculty, Gdańsk University of Technology (GUT), 11/12 G. Narutowicza St., 80-233 Gdańsk, Poland; magfabja@student.pg.edu.pl; 2 Department of Analytical Chemistry, Chemical Faculty and BioTechMed Center, Gdańsk University of Technology (GUT), 11/12 G. Narutowicza St., 80-233 Gdańsk, Poland; 3 Department of Analytical Chemistry, Chemical Faculty and EcoTech Center, Gdańsk University of Technology (GUT), 11/12 G. Narutowicza St., 80-233 Gdańsk, Poland; martobis@pg.edu.pl

**Keywords:** disposable baby diapers, phthalate, analytical procedures, TOPSIS, MCDA, green analytical chemistry, environmental impact

## Abstract

This study presents the application of one of the tools from the multicriteria decision analysis set (MCDA), the Technique for Order Preference by Similarity to Ideal Solution (TOPSIS). Selected green analytical chemistry metrics were used to rank analytical procedures for the phthalate determination in disposable baby diapers. Nine analytical procedures were assessed in order to find one that has the lowest environmental impact and the best analytical figures of merit. Nine different criteria, where weighting was based on the experts’ evaluation, were used in the procedures’ assessment. With the use of TOPSIS, an easy and straightforward technique, selection of the most appropriate procedure was made.

## 1. Introduction

Phthalates are a group of compounds derived from the esters of 1,2-dibenzene dicarboxylic acid. Due to their properties, they are widely applied in the industry as plasticizers (addition of phthalates increases the flexibility and workability of high molecular weight polymers), heat-transfer fluids and carriers (thanks to their low melting point and high boiling point) [1]. 

The most commonly used phthalates are di(2-ethylhexyl) phthalate (DEHP) and the primary metabolite mono-(2-ethylhexyl) phthalate (MEHP). However, di(n-butyl) phthalate (DBP), diethyl phthalate (DEP), butyl-benzyl phthalate (BBP) and di(n-octyl) phthalate (DnOP) are also abundant [2]. Phthalates are present in ink, paint, adhesives, vinyl flooring, food packaging, furniture, toys, cosmetics and pharmaceuticals, as well as in sanitary products [1,3]. 

There is a growing concern regarding the adverse effect of phthalates on human health. There are several studies presenting the link between human exposure and certain disease occurrences, e.g., cancer, abnormal development and thyroid function, fertility disturbances, type 2 diabetes and cardiovascular disorders. Moreover, several phthalate metabolites can increase the risk of obesity [3].

When it comes to exposure to phthalates, newborns and toddlers are one of the most vulnerable groups of people due to the fact that up to the age of four, their skin is at risk of prolonged exposure to the compounds present in disposable diapers [4]. Nowadays, disposable baby diapers are filled with superabsorbent material, increasing their softness and overall comfort of use. However, because of that, they can also be a source of volatile organic compounds (VOCs) and endocrine-disrupting chemicals, such as phthalates [5]. It is very important to control and monitor the safety of disposable baby diapers in order to reduce the adverse effect that studied compounds can have on the well-being of diaper-wearing children [5].

To the best of the authors’ knowledge, only a few studies dealing with phthalate determination in disposable baby diapers have been published within the last 10 years, as can be seen in Table 1. However, there has been a significant increase in the number of publications on this topic in the last two years [6,7,8], which indicates the importance of this topic and increased interest in it.

Gas chromatography coupled to mass spectrometry (GC-MS) is the most widely applied technique for phthalate determination in disposable baby diapers and other sanitary products of similar matrices. GC-MS was applied in seven out of nine research articles gathered in Table 2. Due to sufficient volatility of phthalates, there is no derivatization needed. However, due to the complicity of the matrix, there is a need for extraction prior to the chromatographic analysis. According to the published scientific reports, solvent extraction is preferred.

Although there is an increasing number of research articles depicting phthalate determination in different sanitary products, there is a lack of critical comparisons of developed methodologies in terms of both the parameters of analytical figures of merits as well as their green character. It is obvious that modern analytical procedures should be designed keeping in mind both the requirements of green analytical chemistry and the necessity of sustainable development. Multicriteria decision analysis (MCDA) can be applied in the assessment of the environmental impact of different approaches to phthalates determination. In MCDA, a set of tools such as the Preference Ranking Organization METHOD for Enrichment of Evaluations (PROMETHEE) [14] or Technique for Order of Preference by Similarity to Ideal Solution (TOPSIS) [15] are used to rank procedures.

This work aims to fill the gap of knowledge in the field of simultaneous assessment of validation parameters and environmental impact of available procedures for phthalate determination in baby sanitary products. Moreover, the applicability of TOPSIS as the appropriate method selection is described. This case study was aimed at finding a green analytical procedure for phthalate determination in diapers and related product samples. The present study stands as a starting point for future research concerning the safety of newborns and toddlers exposed to the chemicals released from disposable baby diapers. We believe that this work will benefit those who research phthalates in sanitary products and will enable the development of methods with minimal influence on the environment.

## 2. Result and Discussion

### 2.1. Clustering of Variables

**Figure 1:**
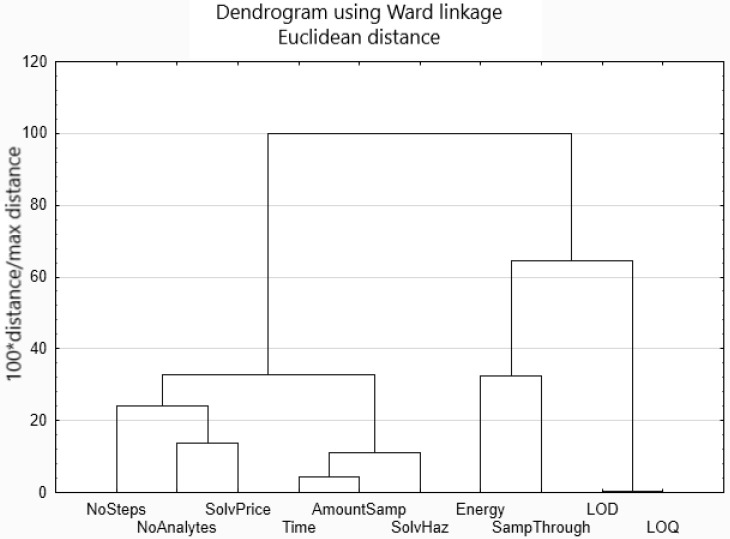
Results of cluster analysis grouping of variables.

Cluster analysis facilitates finding possible correlations between variables and data dimensionality reduction. According to the results (Figure 1), three clusters can be identified (the cut-off was set to 60% of the distance, to a maximum distance ratio from 10 criteria).

The obtained results from the clustering of variables are as follows:

i. There are several correlations between selected variables observed: LOD and LOQ can be further treated as a single variable. The relation between LOD and LOQ is the following: LOQ = 3 × LOD, which was calculated either by the authors of the investigated procedures or by the authors of this study. Therefore, the resulting determination coefficient R^2^ = 1.
ii. Time and amount of sample are correlated, which indicates that more time is needed for the preparation and analysis of a larger sample. Correlation is affected mostly by Procedure 9, which is an outlier, described in more detail later;
iii. Solvent hazard variability is correlated with two former variables. It indicates that a large number of solvents are needed to extract analytes from large samples;
iv. The price of solvents is correlated with the number of analytes, which is an indication that multianalyte procedures require larger inputs in terms of solvents;
v. The number of analytical steps is correlated with two former variables. Thus, it can be concluded that more analytical steps are needed to deal with multianalyte situations. On the other hand, a higher number of steps requires higher solvent contribution;
vi. Both groups (time, amount of sample and solvent hazards, as well as solvent price, number of analytes and number of steps) are loosely correlated;
vii. The last cluster consists of energy and sample throughput. Sample throughput measures the number of analyses that can be performed within one hour, which is related to the energy consumption. The higher the sample throughput, the less energy is needed for a single analysis.

### 2.2. Clustering of Analytical Procedures

**Figure 2:**
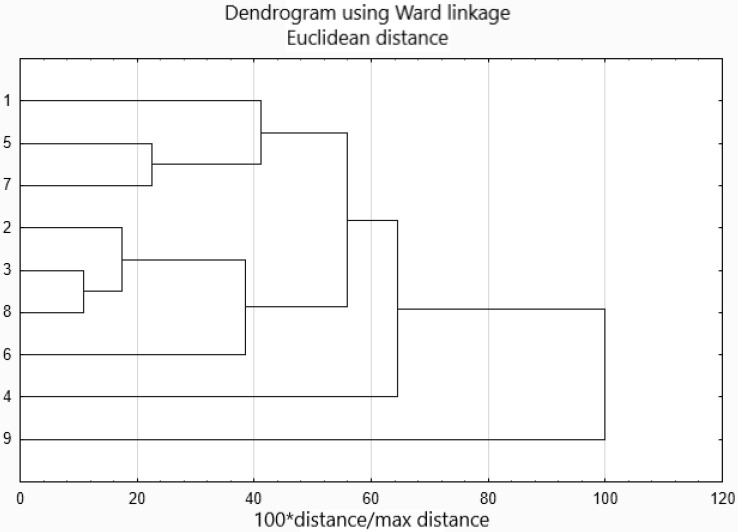
Cluster analysis results of analytical procedures grouping.

According to the cluster analysis (Figure 2), the clustering of objects shows the following patterns:

i. Procedures 3 and 8 are more loosely related to 2;
ii. Procedures 5, 7 and 1 are also somehow related;
iii. Procedure 9 is an outlier.

Going in-depth, the first cluster, which gathers procedures 3, 8 and 2, clusters methodologies based on the same analytical technique; procedures 3 and 8 use gas chromatography coupled to mass spectrometry (GC-MS), while procedure 2 uses gas chromatography with flame ionization detector (GC-FID). However, all of them are characterized by a similar amount of solvent used, the same number of procedures steps and similar energy consumption.

The second cluster grouped procedures 5 (based on the liquid chromatography coupled to tandem mass spectrometry (LC-MS/MS), 7 (based on GC-MS) and 1 (DART-MS/MS). All of them are characterized by the same number of procedure steps and similar energy consumption.

Procedure 9 is an outlier since the amount of solvent used during the analysis was ten times higher than in the rest of the analytical procedures described before. This procedure significantly differs from the others in terms of solvent hazards and time of analysis (six times longer).

### 2.3. Ranking

The application of TOPSIS requires setting the ranking of the weights of all the variables (gathered in Table 1). The weights were applied based on the variable’s importance in the context of green chemistry and analytical figures of merit. Thus, LOQ is given a very low weight as all analytical procedures have satisfactory LOQ. The number of samples is of little importance as diapers are not very expensive and easy to obtain. Reagent price is given relatively low weight as it is within a satisfactory range. The number of steps is given the same weight of 0.05 due to little variability. The highest weight is given to solvent hazards as it is the most influential factor of the analytical procedure’s greenness assessment. The number of analytes and two time-related criteria are given high weight values, as all criteria describe the amount of analytical information obtained in a reasonable unit of time. Energy consumption is given a moderate weight as it should not be minimized but is not critical in terms of optimization of analytical methodologies.

The ideal methodology is characterized by the best parameters that could be achieved [16]. After the application of TOPSIS ranking (Table 3), it can be seen that procedure 1, DART-MS/MS, is the closest to the ideal analytical procedures. Direct analysis in real-time does not require the sample preparation step and there is no solvent usage, which is a significant advantage, resulting in the highest place in the ranking. Second, achieving a 0.677 score, is a procedure based on the liquid chromatography coupled to tandem mass spectrometry (procedure 6). The least ideal solution and the last place in the ranking is dedicated to the outlier—procedure 9. This is not a surprising result since the use of this procedure takes six times longer than the others. Furthermore, it obtained the highest solvent hazard score.

## 3. Materials and Methods

### 3.1. Dataset Creation

The dataset used for TOPSIS analysis was collected from the research papers published in the Web of Science, Scopus, Science Direct and Nature databases. Specific keywords like phthalate, disposable baby diapers, and newborns were applied in order to find publications of interest. Due to the matrix similarity, sanitary pads, wipes and toilet tissue paper were also taken under consideration.

### 3.2. Cluster Analysis

Cluster Analysis (CA) belongs to a set of multivariate statistical tools that allow for grouping objects and variables according to their similarity. Briefly, the unsupervised algorithm finds an internal pattern in the set of data with no previous considerations on the dataset. The similarity (or dissimilarity) of objects or variables is calculated on the basis of correlation or distance. The grouping of both variables and objects is performed with the Euclidean distance and cluster formation method after Ward. After standardization of the initial dataset, CA calculations are performed with Statistica12 software. More details about the algorithm may be found elsewhere [17].

### 3.3. TOPSIS

One of the frequently applied MCDA algorithms is TOPSIS. This is an expert system that is applied for decision making and was developed in 1981 by Hwang and Yoon [18]. The analysis leads to the final ranking of all alternatives; as a result, the selection of the best option among all available is easily possible. The alternative that has the shortest distance to the positive ideal solution and the farthest distance to the negative ideal solution at the same time is the winner.

The assessment procedure for MCDA can be performed in a few simple steps. In the beginning, the main aim of the analysis should be clearly stated. This case study is aimed at finding a green analytical procedure for phthalate determination in diapers and related product samples. The dataset prepared for application in MCDA methodology consists of criteria and alternatives, which should be clearly defined. Generally, criteria are parameters that describe the set of alternatives and make the evaluation easy and systematic. Alternatives are available options that allow reaching the goal stated in the aim of the MCDA study. All the alternatives must be described by criteria expressed with numerical values or at least they must be easily transformable into numbers. In the next step of MCDA assessment, the assigning of proper weight values to each criterion is done. This means that there is a possibility to differentiate the importance of criteria.

The last step is the application of the TOPSIS algorithm. Its mechanism can be described in several steps, as described in other papers [19]. All TOPSIS calculations were performed in the Excel program (Microsoft 2016).

### 3.4. The Dataset

Based on the literature reports available in the Web of Science and Scopus databases, nine analytical methodologies were found and applied to a given research problem. All of them were described by certain variables, which were used to set the criteria for clustering analysis in order to find parameters that are the most significant in choosing the best methodology. In this context, the term “the best” corresponds to the methodology, which is described by the most relevant analytical figures of merit as well as meets the requirements of green analytical chemistry. Thus, ten criteria were established in order to perform the first grouping:

- number of steps necessary to perform the analysis;
- number of analytes determined in the single run;
- the solvent prize, taken from the website for the Polish market, with prices recalculated based on the price for 1 L;
- the time needed to perform the analysis (extraction time + time of chromatographic analysis);
- amount of sample used for the analysis;
- solvent hazards, which was calculated according to [20];
- energy consumption during a single run;
- sample throughput calculated as 60 min divided by the time of chromatographic analysis;
- limit of detection (LOD) and limit of quantification (LOQ), calculated as 3.3 × LOD.

Such a selection of variables comprehensively covers many aspects of the procedure assessment. They deal with the most basic metrological parameters but also refer to many green analytical chemistry aspects (solvents applied, energy consumed) and general performance (number of analytes, analysis time, number of procedural steps, analytical throughput) of the procedure.

All the data gathered for the MCDA and CA analyses are presented in Table 3.

## 4. Conclusions

In this study, TOPSIS was applied for the selection of the most relevant analytical procedure for the phthalate determination in disposable baby diapers. According to data analysis and procedures rank, the procedure that gained the best result was the direct analysis in real-time (DART) coupled to mass spectrometry (MS) technique. The lack of sample preparation is a crucial advantage of the given technique. Apart from the low impact on the environment, good metrological parameters were observed. Multicriteria decision analysis is a useful approach for the selection of procedures meeting the green analytical chemistry requirements.

## Figures and Tables

**Table 1:** Dataset characteristics.

Criteria	Analytical Procedures								
	1 [9]	2 [10]	3 [11]	4 [6]	5 [7]	6 [12]	7 [5]	8 [13]	9 [8]
**Analytical methodology abbreviation**	DART-MS/MS	MSPE/GC-FID	GC-MS	GC-MS	GC-MS	PLE/GC-MS	LC-MS/MS	GC-MS	GC-MS
**LOQ [mg/L]**	0.001485	0.00066	0.1	0.7689	0.33	0.02376	0.0001	0.002	0.001188
**LOD [mg/L]**	0.00045	0.0002	NI	0.233	0.1	0.0072	NI	NI	0.00036
**Reagent (list of all reagents used in the procedure)**	direct analysis	6 mL HCl	~10 mL DCM	83 mL hexane, 47 mL acetone	0.5 mL Milli-Q water. 10 mL hexane. 3 mL MeOH	100 mL ethyl acetate. 20 mL methanol	6 mL methanol	5 mL hexane	102.2 mL DCM, 154.8 mL hexane
**Solvent hazards**	0	0	598	6878.4	861.1	1044	94.2	407	18,712.28
**Reagent price (euro)**	-	0.18	0.22	3.61	0.37	5.56	0.15	0.14	8.78
**Amount of sample (g)**	0.9985	0.1	0.05	0.5	0.3	5.25	0.435	0.3	100
**Number of other analytes**	21	4	7	15	8	54	4	24	57
**Time of analysis (min)**	few sec.	48	NI about the extraction time + 37.5	86	138.5	31	36	118.66	992
**Sample throughput**	few sec.	3	1	2	1	2	2	1	1
**Procedure steps**	2	3	3	3	2	3	2	3	4
**Energy consumption (kWh)**	3.2	1.6	2.55	1.77	4.03	1.77	3.2	2.62	2.6

**Table 2:** Weights applied for TOPSIS according to the scenario.

	Scenario
LOQ	0.01
Solvent hazards	0.28
Reagent price	0.05
Amount of sample	0.01
Number of other analytes	0.2
Time of analysis	0.15
Sample throughput	0.15
Procedure steps	0.05
Energy consumption	0.1

**Table 3:** Ranking results of analytical procedures according to a given scenario.

Scenario	
Procedure Number	Similarity to an Ideal Solution
1	0.799
6	0.677
8	0.638
2	0.616
7	0.612
3	0.609
5	0.599
4	0.521
9	0.268

## Data Availability

Data is contained within this article.
